# Enhanced Optoelectronic Synaptic Performance in Sol–Gel Derived Al-Doped ZnO Thin Film Devices

**DOI:** 10.3390/ma18132931

**Published:** 2025-06-20

**Authors:** Dabin Jeon, Seung Hun Lee, Sung-Nam Lee

**Affiliations:** 1 Department of IT & Semiconductor Convergence Engineering, Tech University of Korea, Siheung 15073, Republic of Korea; 2 Department of Semiconductor Engineering, Tech University of Korea, Siheung 15073, Republic of Korea

**Keywords:** ZnO, AZO, sol–gel, optoelectronic, synapse, neuromorphic

## Abstract

We report the fabrication and characterization of Al-doped ZnO (AZO) optoelectronic synaptic devices based on sol–gel-derived thin films with varying Al concentrations (0~4.0 wt%). Structural and optical analyses reveal that moderate Al doping modulates the crystal orientation, optical bandgap, and defect levels of ZnO films. Notably, 2.0 wt% Al doping yields the widest bandgap (3.31 eV), stable PL emission, and uniform deep-level absorption without inducing significant lattice disorder. Synaptic performance, including learning–forgetting dynamics and persistent photoconductivity (PPC), is strongly dependent on Al concentration. The 2.0 wt% AZO device exhibits the lowest forgetting rate and longest memory retention due to optimized trap formation, particularly Al–oxygen vacancy complexes that enhance carrier lifetime. Visual memory simulations using a 3 × 3 pixel array under patterned UV illumination further confirm superior long-term memory (LTM) behavior at 2.0 wt%, with stronger excitatory postsynaptic current (EPSC) retention during repeated stimulation. These results demonstrate that precise doping control via the sol–gel method enables defect engineering in oxide-based neuromorphic devices. Our findings provide an effective strategy for designing low-cost, scalable optoelectronic synapses with tunable memory characteristics suitable for future in-sensor computing and neuromorphic vision systems.

## 1. Introduction

As modern computing demands grow increasingly complex, conventional von Neumann architectures face critical challenges, including high energy consumption, data transfer latency, and limited parallelism. To overcome these bottlenecks, neuromorphic systems inspired by biological neural networks have attracted increasing interest [1,2,3]. These systems aim to replicate the brain’s learning and memory capabilities through hardware implementations of artificial synapses and neurons. Among various approaches, optoelectronic synaptic devices have emerged as promising platforms for next-generation in-sensor computing. These devices modulate their electrical conductance in response to optical stimuli [4,5,6]. By integrating sensing, memory, and processing functions in a single device, they enable real-time, energy-efficient data handling [7,8]. This capability is particularly advantageous for applications, such as visual recognition, adaptive image processing, and edge artificial intelligence (AI), where low latency and power efficiency are essential [9,10]. Optoelectronic synapses rely on light-triggered changes in electrical conductance to emulate biological synaptic behaviors, such as short-term plasticity (STP), long-term potentiation (LTP), paired-pulse facilitation (PPF), and memory retention [11,12,13]. The performance and stability of these functions are highly dependent on the active material, which must offer strong light sensitivity, tunable electronic properties, and stability under repeated switching.

To this end, many materials have been explored for optoelectronic synaptic systems. These include two-dimensional semiconductors such as MoS_2_, graphene oxide, halide perovskites, organic semiconductors, and various metal oxides [11,13,14,15,16]. Two-dimensional semiconductors offer high carrier mobility and strong light–matter interactions, but their atomically thin nature often leads to low absorption and integration challenges. Halide perovskites exhibit excellent optoelectronic properties and long carrier lifetimes; however, they suffer from environmental instability and lead toxicity concerns. In contrast, zinc oxide (ZnO) is particularly appealing due to its wide bandgap (3.37 eV), high exciton binding energy (60 meV), chemical stability, and strong ultraviolet (UV) sensitivity [6,17]. In addition, ZnO exhibits intrinsic n-type conductivity owing to intrinsic defects, such as oxygen vacancies and interstitial Zn [18], which are favorable for optically driven charge modulation in synaptic devices. ZnO thin films can be synthesized using various deposition techniques, including radio-frequency (RF) sputtering, chemical vapor deposition (CVD), pulsed laser deposition (PLD), molecular beam epitaxy (MBE), atomic layer deposition (ALD), and sol–gel processing [17,18,19,20,21]. Among these, the sol–gel method stands out for its low cost, scalability, compatibility with large-area substrates, and low-temperature processing [13,17]. Notably, it offers excellent control over precursor chemistry and post-annealing conditions, enabling fine-tuning of carrier concentration and defect density. Despite these advantages, pristine ZnO films prepared by the sol–gel method often exhibit limited conductivity and slow photoresponse due to grain boundary scattering and trap-assisted recombination [22,23]. To overcome these limitations, group III element doping, particularly aluminum (Al), has been widely adopted [24,25]. Al^3+^ ions substitute for Zn^2+^ in the lattice, increasing free carrier concentration and thereby enhancing electrical conductivity [24,25,26]. Additionally, Al doping introduces deep-level trap states such as Al–oxygen vacancy complexes [26]. These defects can prolong photocarrier lifetimes by suppressing recombination, thus improving persistent photoconductivity (PPC)—a key mechanism for memory retention in synaptic devices [26]. While previous studies have demonstrated that Al doping can improve ZnO performance, the optimal doping concentration for balancing enhanced conductivity, PPC behavior, and synaptic function remains underexplored in sol–gel processed films. In this study, we report this gap by systematically investigating the effects of Al concentration on the structural, optical, and neuromorphic characteristics of Al-doped ZnO (AZO) optoelectronic synaptic devices. Our results reveal a clear correlation between doping level, defect states, and synaptic behavior, enabling the identification of an optimal doping level for robust and efficient optoelectronic synaptic performance.

## 2. Materials and Methods

Al-doped ZnO (AZO) thin films were deposited via a sol–gel spin-coating technique on c-plane sapphire substrates, chosen for their excellent lattice compatibility and thermal stability. The precursor solution was prepared by dissolving 0.025 mol zinc acetate dihydrate (ZAD) and 0.025 mol monoethanolamine (MEA) in 50 mL 2-methoxyethanol, maintaining a 1:1 molar ratio. This corresponds to a precursor molarity of 0.5 M, which was optimized to ensure uniform film formation and consistent doping levels. Aluminum nitrate nonahydrate was added at concentrations of 0, 1, 2, and 4 wt% relative to ZAD to investigate the effects of Al doping on synaptic behavior. The solution was stirred at 1000 rpm and 100 °C for 30 min to ensure homogeneity. The resulting sol was spin-coated onto the substrate using five drops at 6000 rpm for 30 s. Spin-coating at this speed was selected to achieve uniform thin film morphology with high surface coverage and reproducibility. The pre-baking temperature and duration were optimized to evaporate volatile components without initiating premature crystallization [13,17]. Each layer was pre-baked at 200 °C for 5 min to remove solvents. Post-deposition, rapid thermal annealing was performed at 900 °C for 1 min in air to improve crystal properties and activate dopants. The high-temperature thermal annealing step was specifically chosen to facilitate grain growth, enhance Al^3+^ substitution into the Zn^2+^ lattice sites, and reduce defect densities such as interstitials and hydroxyl groups. The short annealing minimized film damage and preserved interface integrity, which is critical for stable optoelectronic and synaptic behavior [24]. The final AZO film thickness was approximately 100 ± 5 nm, as measured by alpha step. The fabricated AZO films served as the active semiconductor layer in metal–semiconductor–metal (MSM) structured optoelectronic synaptic devices. A standard photolithographic process was used to define the device geometry, with a sensing area of 50 × 825 μm^2^. Aluminum (Al) electrodes, 50 nm thick, were deposited via thermal evaporation under high vacuum to form symmetric MSM contacts along the long axis of the sensing region. This simple and reproducible process enabled the formation of high-quality MSM devices suitable for UV-stimulated synaptic evaluation. The resulting structure allowed for stable excitatory post-synaptic current (EPSC) responses under UV illumination, effectively mimicking biological synaptic functions.

The optoelectronic and synaptic behaviors of the AZO thin film devices were systematically characterized using electrical and optical measurement techniques. The optical properties, including bandgap estimation, were assessed via UV–visible absorption spectroscopy (Thermo Fisher Scientific, Evolution 300, Waltham, MA, USA) and photoluminescence (PL, (Dongwoo, Seoul, Republic of Korea)) analysis. Crystallographic characteristics were investigated using X-ray diffraction (XRD, Rigaku Corporation, Tokyo, Japan) and defect-related vibration modes were analyzed using Raman spectroscopy (Dongwoo, Seoul, Republic of Korea). Electrical measurements were performed using an HP4155A (HP 4155A, Santa Rosa, CA, USA) semiconductor parameter analyzer. Both dark current and UV-induced photocurrent were recorded under 365 nm UV illumination provided by a calibrated UV LED. Optoelectronic synaptic behavior was evaluated by monitoring the EPSC at a constant bias voltage of 1.0 V. Optical potentiation was induced by pulsed UV stimulation, resulting in a gradual increase in EPSC, followed by a natural depression phase where EPSC decayed after the cessation of light.

## 3. Results and Discussion

### 3.1. Optical and Structural Characterization of AZO (0–4.0 wt% Al) Thin Films on Sapphire Substrates

**Figure 1:**
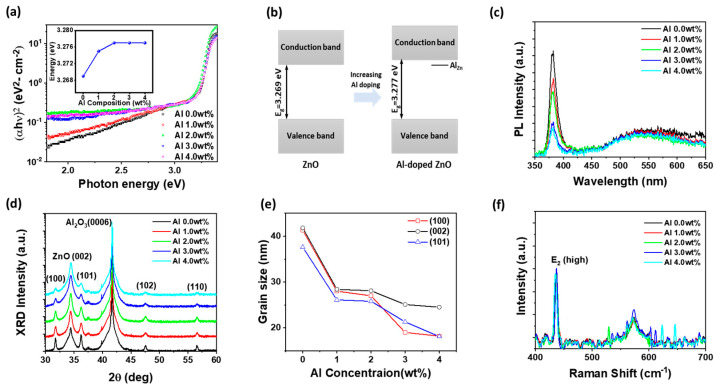
(**a**) Optical absorption spectra, (**b**) schematic energy band diagrams of ZnO and AZO films, (**c**) photoluminescence spectra at room temperature, (**d**) X-ray diffraction θ/2θ scan for crystallographic structure, (**e**) grain size of the (100), (002), and (101) ZnO planes as a function of Al concentrations in AZO film, and (**f**) Raman spectroscopy for vibrational mode analysis of Al-doped ZnO thin films with varying Al concentrations. Inset of (**a**) shows the optical bandgap analysis for Al-doped ZnO films using Tauc plots.

The optical properties of sol–gel-derived AZO thin films were systematically investigated using UV–visible absorption and room-temperature PL spectroscopy, with Al concentrations ranging from 0 to 4.0 wt%. As shown in Figure 1a, Tauc plot analysis of the absorption spectra revealed that the optical bandgap increased slightly from 3.269 eV for undoped ZnO to 3.277 eV at an Al doping concentration of 2.0 wt%, and then remained saturated at 3.277 eV even with further doping up to 4.0 wt%, as shown in the inset of Figure 1a. This initial increase is attributed to the Burstein–Moss effect, in which the substitution of zinc ions with smaller aluminum ions leads to an increase in free electron concentration and a corresponding upward shift in the Fermi level [27]. The saturation in bandgap widening beyond 2.0 wt% is likely due to the onset of band tailing and defect state formation, which compensate for further shifts by introducing localized states near the conduction band edge. In the sub-bandgap region (2.0–3.0 eV), the absorption behavior also showed a clear dependence on Al content. Films with 0 and 1.0 wt% Al exhibited gradually decreasing absorption, indicative of a low density of trap states. In contrast, the 2.0 wt% Al-doped film displayed a relatively flat and sustained absorption profile, suggesting the formation of uniformly distributed, optically active defect states that enhance photoexcitation and prolong carrier lifetimes. At Al concentrations of 3.0 and 4.0 wt%, the sub-bandgap absorption remained strong but became increasingly broad and featureless, indicating reduced spectral selectivity. This behavior is attributed to the formation of disordered defect states, such as interstitial Al and defect clusters, which blur the distinction between discrete energy levels [28]. These structural irregularities introduce a continuum of localized states, allowing photon absorption across a wide energy range. To further illustrate these electronic changes, Figure 1b presents schematic energy band diagrams for the undoped ZnO and 4.0 wt% Al-doped AZO films, incorporating the energy levels of Al-related defect states, such as interstitial Al, located below the conduction band. These diagrams reflect the optical trends observed in Figure 1a and highlight the role of defect-induced sub-bandgap states in modifying absorption behavior. Additionally, the prevalence of non-radiative recombination centers at high doping levels diminishes radiative transitions, thereby reducing optical efficiency despite elevated absorption. Figure 1c shows the room-temperature PL spectra of the Al-doped ZnO thin films. As the Al concentration increased, the band-edge PL intensity continuously decreased, indicating an increase in non-radiative recombination pathways caused by structural disorder and defect-induced carrier trapping [25,28,29]. Interestingly, the band-edge peak position remained nearly unchanged up to 2.0 wt% Al, measured at 380.1 nm for both undoped ZnO and 1.0 w% Al, and slightly redshifted to 381.4 nm at 2.0 wt%, despite the simultaneous increase in optical bandgap observed in the absorption spectra (Figure 1a). This discrepancy arises from the different physical mechanisms underlying the two measurements: while the Tauc-derived bandgap reflects interband transitions influenced by the Fermi level shift (i.e., the Burstein–Moss effect), PL is governed by radiative recombination dynamics, which are more sensitive to localized states and relaxation processes [27,28,29]. The observed slight redshift at 2.0 wt% may originate from increased carrier localization or deep-level trap-assisted recombination, which slightly lowers the radiative recombination energy despite the widened bandgap. At higher doping levels (≥3.0 wt%), a slight blue shift in the PL peak was observed, 379.2 nm at 3.0 wt% and 378.5 nm at 4.0 wt%, indicating a transition toward more direct band-edge recombination as defect-related deep levels become saturated or inactive due to excessive doping. Despite the drop in PL intensity, the position of deep-level emission around 550 nm remained relatively constant across all doping concentrations. This indicates that Al incorporation, even at higher levels, does not significantly alter the density of intrinsic deep-level defects such as oxygen vacancies or interstitial zinc atoms [28]. These PL characteristics are consistent with the optical absorption data, which showed enhanced sub-bandgap absorption with increasing Al content, particularly in the visible range, suggesting the presence of non-radiative defect states that contribute to broad absorption but do not enhance radiative emission. The 2.0 wt% Al-doped ZnO film, which demonstrated the highest UV absorbance below 380 nm, offers the best trade-off between efficient UV harvesting, band structure modulation, and minimized non-radiative loss, making it the most suitable candidate for optoelectronic synaptic applications requiring both sensitivity and stability. High-resolution X-ray diffraction (HR-XRD) θ/2θ scans were performed to examine the crystallographic properties of ZnO thin films synthesized by the sol–gel method, as shown in Figure 1d. The diffraction patterns revealed five characteristic peaks corresponding to the (100), (002), (101), (102), and (110) planes of the hexagonal wurtzite ZnO structure [25]. Among these, the (002) peak was dominant, indicating a strong preferential orientation along the c-axis perpendicular to the substrate surface. This result suggests that the sol–gel process promotes oriented crystallite growth along the (002) direction while still incorporating minor contributions from other planes. With increasing Al doping concentration, the intensities of the (100), (101), (102) and (110) peaks gradually decreased, while the (002) peak intensity significantly increased, indicating an enhanced preferential orientation along the c-axis. However, the simultaneous broadening of the (002) peak with increasing Al content, as evidenced by the increased full width at half maximum (FWHM), suggests that although local (002) orientation may be enhanced in some grains, the overall crystallinity is compromised. Crystallite sizes were estimated using the Scherrer equation (D = Kλ/βcosθ), where D is the crystallite size, λ is the X-ray wavelength, β is the FWHM of the peak, and θ is the Bragg angle. As shown in Figure 1e, the grain sizes of the (100), (002), and (101) planes were estimated from the XRD data in Figure 1d using the Scherrer equation. With increasing Al concentration from 0 to 4.0 wt%, the grain size of all orientations decreased significantly. For example, the (100) and (002) grain sizes dropped from over 40 nm at 0 wt% to below 20 nm at 4.0 wt%. This reduction indicates that Al doping inhibits crystallite growth and induces finer microstructures, which may lead to increased grain boundaries, influencing carrier transport and enhancing trap-assisted optoelectronic synaptic performance. This coexistence of increased (002) intensity and broader peaks can be attributed to localized alignment of grains along the c-axis, while Al incorporation induces lattice strain, grain boundary formation, and microstructural inhomogeneity. Such structural evolution increases defect density and limits coherent grain growth, which can degrade charge carrier mobility and promote trap-assisted recombination. Therefore, although the (002) orientation appears enhanced statistically, the microstructural quality is reduced, influencing carrier transport and trap state distributions. These factors are critical in determining the performance of optoelectronic synaptic devices, particularly in terms of EPSC dynamics and long-term retention. These results indicate that while Al doping improves the degree of (002) orientation, it also introduces microstructural inhomogeneity, which may influence carrier transport and trap state distribution in optoelectronic synaptic applications.

Complementary insights into lattice dynamics were obtained from Raman spectroscopy, as presented in Figure 1f. All samples exhibited the E_2_(high) mode (~437.20 cm^−1^ at 0 wt%), a characteristic vibration of the wurtzite ZnO structure associated with nonpolar in-plane oscillation of oxygen atoms [30]. As the Al content increased, the position of the E_2_(high) mode exhibited a slight redshift z: 437.12 cm^−1^ (1.0 wt%), and 436.97 cm^−1^ (2.0 wt%), while remaining relatively close to the undoped value. This trend indicates that the ZnO lattice structure was largely preserved under moderate doping conditions. However, at 3.0 and 4.0 wt%, the E_2_(high) peak shifted to lower wavenumbers (436.22 cm^−1^ and 435.31 cm^−1^, respectively) suggesting the onset of tensile strain or increased structural disorder due to excessive dopant incorporation [30,31]. This trend in Raman shift values with Al content further supports the XRD analysis, reinforcing the conclusion that 4.0 wt% doping induces significant strain and disorder. This redshift is indicative of lattice relaxation or distortion, potentially caused by interstitial Al atoms or the formation of Al–O vacancy (Al–V_O_) complexes that disrupt the periodic potential [26,31]. This trend is consistent with the XRD results, where the (002) diffraction peak intensity increased with Al doping, suggesting enhanced c-axis orientation, but the FWHM also broadened, pointing to reduced crystalline uniformity at higher Al concentrations. Additionally, the enhancement of vibrational features in the 550–600 cm^−1^ range, assigned to longitudinal optical modes A_1_(LO) and E_1_(LO), further confirms the accumulation of defect-related scattering at higher doping levels. Overall, the results indicate that Al doping up to 3.0 wt% preserves the ZnO lattice with minimal strain, while excessive doping at 4.0 wt% introduces disorder and defect states.

### 3.2. Electrical and Persistent Photoconductivity Characteristics of Al-Doped ZnO Devices

**Figure 2:**
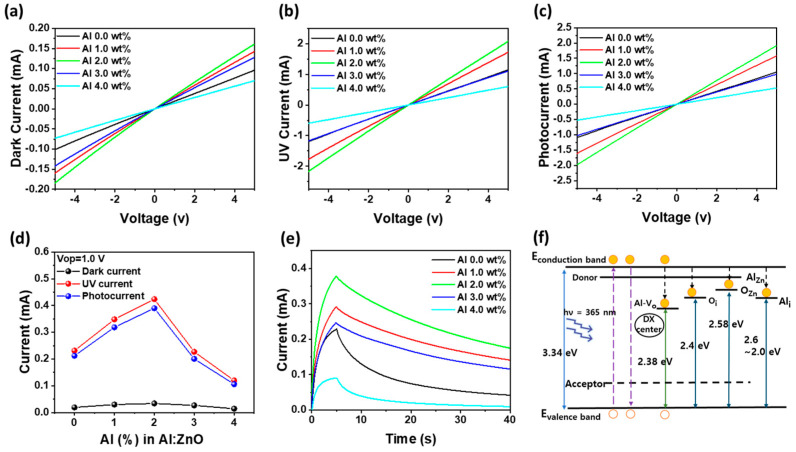
Current–voltage (I–V) characteristics of Al/AZO/Al optoelectronic devices under (**a**) dark and (**b**) UV (365 nm) illumination conditions with varying Al doping concentrations. (**c**) Extracted photocurrent (I_ph_ = I_UV_ − I_dark_) as a function of applied bias from −5.0 V to + 5.0 V. (**d**) Comparison of dark current, UV current, and photocurrent at a fixed bias of 1.0 V as a function of Al concentration. (**e**) Time-resolved photocurrent response of the devices under repeated UV on/off cycling (5 s on, 35 s off per cycle). (**f**) Schematic energy band diagram of AZO films illustrating Al-induced defect states, including deep-level traps and DX centers, that contribute to PPC. The involvement of deep-level traps such as Al–oxygen vacancy complexes and DX-like centers is inferred based on the literature [26,27,28].

Figure 2a presents the current–voltage (I–V) characteristics of AZO thin film-based Al/AZO/Al optoelectronic devices under dark and UV (365 nm) illumination conditions, with varying Al doping concentrations (0, 1.0, 2.0, 3.0, and 4.0 wt%). The graphs include (a) dark current, (b) UV-illuminated current, (c) photocurrent (UV–dark), and (d) extracted photocurrent as a function of Al content. In Figure 2a, the dark current increases gradually with increasing Al doping. This trend is attributed to enhanced carrier concentration due to Al^3+^ substituting Zn^2+^ in the ZnO lattice, introducing additional free electrons [25,26]. As the Al doping level increases from 0 wt% to 2.0 wt%, the electron concentration of the AZO film increases significantly from 3.1 × 10^17^/cm^3^ to 2.2 × 10^18^/cm^3^. This increased electron density directly enhances electrical conductivity, thereby boosting the dark current. This carrier concentration peak at 2.0 wt% plays a critical role in improving not only the baseline conductivity but also the responsiveness of the device to optical stimuli. However, further increasing Al doping to 4.0 wt% leads to a decrease in electron concentration (to 1.05 × 10^18^/cm^3^), suggesting that over-doping introduces structural disorder or compensating defects that reduce effective carrier density. As a result, the dark current at 4.0 wt% saturates or slightly declines. Figure 2b shows I–V curves under UV illumination. The UV-induced current increases with Al content up to 2.0 wt%, which reflects the synergistic effects of enhanced carrier density and improved UV absorption. At ≥3.0 wt%, the UV current slightly drops, indicating that excessive Al induces recombination centers or grain boundary scattering, which hinder charge collection. In Figure 2c, the photocurrent, defined as the difference between UV and dark current, shows the same doping-dependent trend. It increases sharply with Al content, peaking at 2.0 wt%, where the combination of high carrier density and strong UV absorption yields maximal photoresponse. Beyond 2.0 wt%, the photocurrent declines due to structural degradation and increased carrier trapping. Figure 2d quantitatively compares photocurrent as a function of Al concentration under a fixed bias (Vop = 1.0 V), with a clear maximum at 2.0 wt%. Figure 2e presents the time-dependent photocurrent response of Al-doped ZnO thin film-based metal–semiconductor–metal devices under UV illumination. The devices were exposed to 365 nm UV light for 5.0 s, followed by a monitoring period in the dark to evaluate PPC. All samples exhibit a gradual increase in current upon UV exposure, followed by a gradual decay after the light is turned off. Among the samples, the device with 2.0 wt% Al doping demonstrates the highest peak photocurrent and the slowest decay rate, indicating the most prominent PPC behavior. This enhanced PPC performance at 2.0 wt% Al is attributed to several synergistic factors. First, Al^3+^ ions substitute Zn^2+^ in the ZnO lattice, increasing the free carrier concentration and improving electrical conductivity, thereby enabling stronger photoresponse [25,26]. Second, UV–visible absorbance spectra confirm that the 2.0 wt% AZO film exhibits the highest absorption below 380 nm, promoting more efficient generation of electron–hole pairs. Third, the sustained sub-bandgap absorption is attributed to optically active deep-level defects, including singly ionized oxygen vacancies (V_O_^+^), zinc interstitials (Zn_i_), and DX-like centers associated with moderate Al doping. DX-like centers refer to deep-level defect states typically associated with donor impurities such as group-III elements (e.g., Al) in II–VI semiconductors like ZnO. These defects act as metastable donor states that trap photogenerated holes [28], allowing the corresponding electrons to remain in the conduction band. This suppresses recombination and prolongs carrier lifetimes, thereby sustaining PPC [6]. The proposed defect configurations are consistent with prior reports on oxygen vacancy-related PPC in AZO thin films [26], and while direct spectroscopic identification (e.g., XPS, temperature-dependent PL) was not conducted in this study, the PPC trends and correlated electrical and structural data support this interpretation. At higher doping concentrations (≥3.0 wt%), the PPC effect is diminished, likely due to excessive Al incorporation leading to crystallographic disorder, defect clustering, and increased carrier scattering, which together degrade carrier lifetime and mobility. 

Additionally, Al over-doping may cause shallower traps or recombination centers rather than stable deep-level DX centers, reducing the retention of photogenerated carriers. These trap-state assignments are made based on precedent in the literature [26,27,28], as direct spectroscopic validation (e.g., XPS, DLTS, or temperature-dependent PL) was not conducted in this study. Overall, these findings demonstrate that 2.0 wt% Al doping optimally balances carrier concentration, trap state formation, and crystallinity, enabling strong and long-lasting PPC. This mechanism is schematically illustrated in Figure 2f, which conceptually depicts the role of deep-level traps, such as DX-like centers and Al–oxygen vacancy complexes, in prolonging carrier lifetimes by suppressing recombination. Such behavior is particularly advantageous for neuromorphic systems and optoelectronic memory applications that rely on light-modulated charge retention.

### 3.3. Short-Term Synaptic Plasticity and Paired-Pulse Facilitation in Al-Doped ZnO Devices

**Figure 3:**
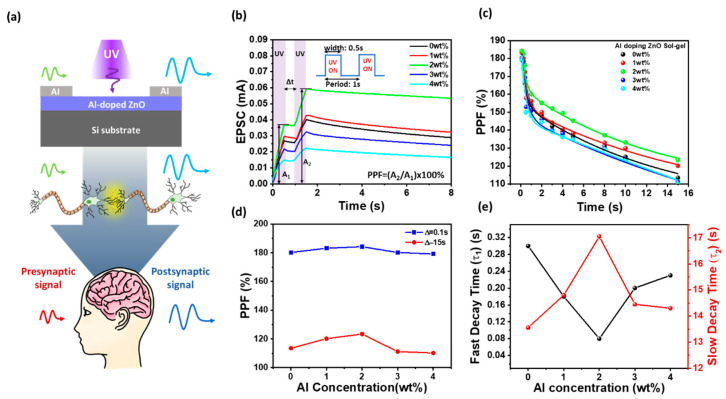
(**a**) Schematic illustration of the Al/AZO/Al optoelectronic synaptic device, indicating UV-induced EPSC generation analogous to a biological synapse. (**b**) PPF behavior under two consecutive UV pulses, where the second EPSC is enhanced due to residual carriers from the first stimulus. (**c**) PPF index as a function of inter-pulse interval (Δt), showing maximum facilitation at Δt = 0.1 s, with the strongest effect observed at 2.0 wt% Al. (**d**) PPF values at Δt = 0.5 s and 15 s plotted as a function of Al concentration, highlighting improved memory retention at moderate doping levels. (**e**) Fast (τ_1_) and slow (τ_2_) decay time constants extracted from the PPF–Δt curves in (**c**), based on a double-exponential fitting model.

Figure 3a exhibits the schematic of the Al/AZO/Al optoelectronic synaptic device showing UV-induced EPSC generation, mimicking a biological synapse. Figure 3b shows the PPF behavior of AZO-based optoelectronic synaptic devices under two successive UV light stimuli. It presents the excitatory post-synaptic current (EPSC) response as a function of time for various Al doping concentrations (0, 1, 2, 3, and 4 wt%). The devices were stimulated twice with UV pulses, separated by different inter-spike intervals (Δt), and the PPF index was extracted based on the ratio of the second EPSC (A_2_) to the first EPSC (A_1_), i.e., PPF = A_2_/A_1_ × 100% [6,11]. As shown in Figure 3b, all devices exhibit a higher second EPSC when the second UV pulse is applied shortly after the first, indicating facilitation behavior analogous to biological short-term synaptic plasticity. The 2.0 wt% Al-doped ZnO device shows the highest amplitude in both initial and subsequent EPSC responses, reflecting superior optical sensitivity and synaptic strength. Figure 3c presents the PPF index as a function of the inter-pulse interval. The PPF index decreases exponentially as the interval increases, which is consistent with the decay of photogenerated carrier trapping and recombination dynamics. Among all compositions, the 2.0 wt% Al-doped AZO device exhibits the highest PPF index across all Δt values, indicating efficient charge accumulation and slow decay of photocarriers, features favorable for mimicking biological temporal learning. In contrast, the 3.0 and 4.0 wt% Al-doped devices show significantly reduced PPF indices, suggesting faster carrier relaxation due to increased grain boundary defects and non-uniform Al incorporation. Figure 3d presents the PPF characteristics of AZO optoelectronic synaptic devices as a function of Al concentration, evaluated at two inter-spike intervals (Δt): 0.1 s (short-term facilitation) and 15 s (long-term facilitation). At Δt = 0.1 s, the PPF index of undoped ZnO was 180.2%, and gradually increased to a maximum of 184.2% at 2.0 wt% Al, followed by a decrease to 179.2% at 4.0 wt%. Similarly, at Δt = 15 s, the PPF index decreased from 180.2% to 113.5% in pristine ZnO, while Al doping led to a peak PPF of 123.4% at 2.0 wt%, and a reduction to 110.2% at 4.0 wt%. Although 2.0 wt% doping exhibited the highest PPF in both short and long Δt cases, the relative enhancement was more pronounced for long-term intervals, suggesting that 2.0 wt% AZO possesses superior charge retention and long-term memory characteristics. Figure 3e further supports these findings by plotting the decay constants (τ_1_ and τ_2_) extracted from the exponential fitting of PPF versus Δt using the following equation [32]:

PPF = A_1_exp(−t/τ_1_) + A_2_exp(−Δt/τ_2_) (1)

Here, τ_1_ represents the fast decay component related to short-term plasticity, while τ_2_ indicates slow decay associated with long-term facilitation. The τ_1_ value was minimized at 2.0 wt%, suggesting rapid initial response, whereas τ_2_ was maximized at the same doping level, indicating prolonged retention of photogenerated carriers. These results demonstrate that 2.0 wt% Al doping offers an optimal balance between carrier generation and trap-assisted retention, thereby enhancing both immediate and persistent synaptic plasticity in AZO-based neuromorphic devices.

### 3.4. UV-Induced Synaptic Plasticity of Al/AZO/Al Optoelectronic Synaptic Devices Under Different Stimulation Conditions

**Figure 4:**
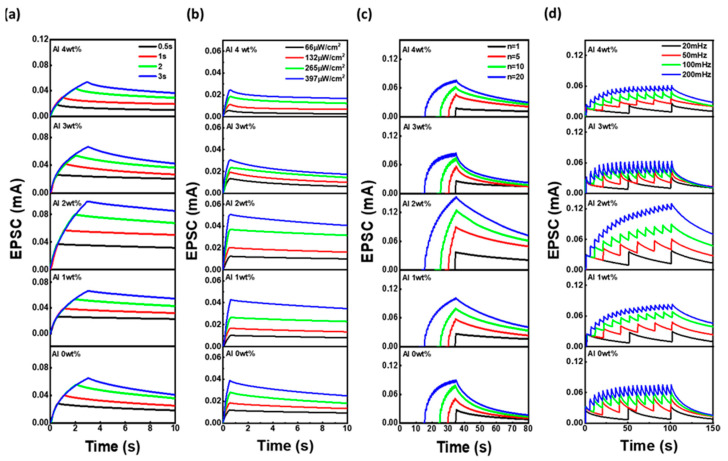
EPSC response and synaptic retention characteristics of Al-doped ZnO optoelectronic synaptic device under various simulation conditions: (**a**) pulse durations (0.5–3 s), (**b**) UV light intensity (66–396 μW/cm^2^), (**c**) the number of UV pulses (1–20 spikes) and (**d**) pulse frequencies (20–100 mHz).

Figure 4 illustrates the synaptic behavior of AZO-based MSM optoelectronic devices under various optical stimulation conditions, with Al concentrations varied from 0 to 4.0 wt%. The influence of light pulse width (0.5–3 s), light intensity (66–397 μW/cm^2^), number of pulses (1–20), and frequency (20–200 mHz) was systematically investigated. In the pulse width-dependent study, increasing the duration of UV illumination enhances carrier generation and accumulation, thereby increasing EPSC. The 2.0 wt% Al-doped ZnO device shows the most pronounced enhancement, suggesting optimal photogenerated carrier trapping and retention. Similarly, when the light intensity increases from 66 to 397 μW/cm^2^, the EPSC increases for all samples, but the 2.0 wt% Al-doped film achieves the highest response. This is attributed to its optimal UV absorption, as confirmed by earlier absorbance measurements, and efficient electron transport due to enhanced conductivity and crystallinity. The frequency-dependent results show that as the stimulation frequency increases (from 20 to 200 mHz), the EPSC summation becomes more prominent due to insufficient recovery time between pulses. At 2.0 wt% Al, the cumulative EPSC effect is maximized, indicating slower recombination kinetics and a favorable trap state distribution that retains photocarriers between pulses. In the pulse number-dependent test, repeated UV inputs from 1 to 20 pulses lead to a gradual EPSC buildup. The 2.0 wt% Al-doped ZnO device demonstrates the highest potentiation and the slowest return to baseline after stimulation, reflecting excellent short-term memory retention. These trends collectively affirm that 2.0 wt% Al offers an ideal balance between electronic and optical properties, such as increased free carrier concentration from substitutional Al^3+^, minimized structural defects, and uniform grain morphology. In contrast, higher Al concentrations (≥3.0 wt%) degrade performance due to excessive defect formation and carrier scattering, weakening synaptic plasticity.

### 3.5. Al Concentration Effect on Learning, Forgetting, and Energy-Efficient Optical Synaptic Plasticity in AZO Optoelectronic Devices

**Figure 5:**
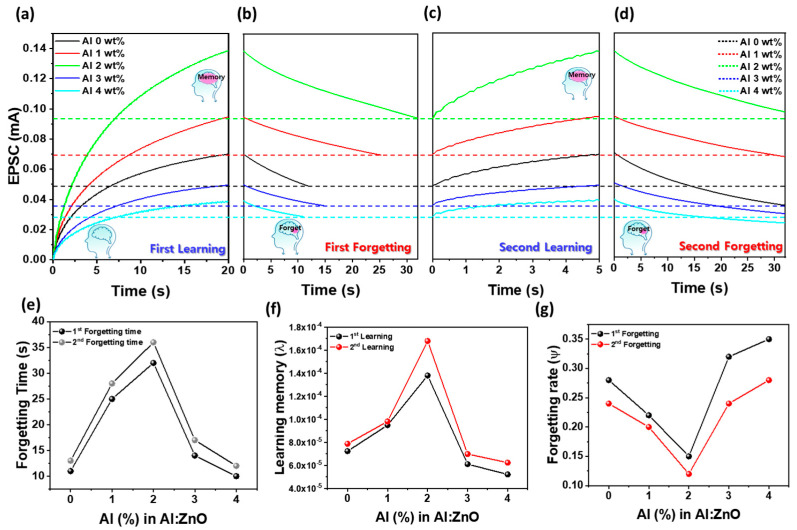
Experience-dependent modulation of synapse behavior in AZO optoelectronic devices with varying Al compositions. (**a**) First learning step represented by the gradual increase in EPSC under repeated UV pulses. (**b**) First forgetting step indicating EPSC decay after termination of UV stimulation. (**c**) Second learning step, initiated from the forgetting threshold, demonstrating memory retention and re-potentiation. (**d**) Second forgetting process following the second learning cycle. (**e**) Comparison of forgetting times between the first and second cycles across different Al concentrations. (**f**) Learning degree (λ) and (**g**) forgetting rate (Ψ) extracted from EPSC dynamics, both systematically modulated by Al doping concentration. The horizontal dashed lines in (**a**–**d**) (black, red, green, blue and cyan) indicate the learning–forgetting threshold, defined as 70% of the maximum EPSC.

Figure 5a–d illustrate the learning and forgetting dynamics of AZO-based optoelectronic synaptic devices with different Al compositions in the ZnO matrix. As the Al content increased from 0 to 4.0 wt%, synaptic plasticity was progressively enhanced, with the most pronounced EPSC response observed at 2.0 wt%. During the learning process, UV light 100 pulses (pulse width: 0.1 s, duty cycle: 50%, power density: 265 µW/cm^2^) were applied, resulting in a gradual increase in EPSC. To evaluate energy consumption, the optical energy (E) absorbed per UV pulse was computed using the following equation [33]:

E = P × A × t × α(2)

where P = 265 μW/cm^2^ is the UV power density, A = 50 μm × 825 μm is the illuminated channel area, t = 0.1 s is the pulse duration, and α = 0.20 is the absorption rate assumed for the AZO film based on reported values for ZnO with different Al compositions [34]. Since Al doping enhances the free carrier concentration and defect-assisted optical transitions, the absorption rate may increase with doping, leading to higher absorption per pulse. Based on this assumption, the minimum energy absorbed per UV pulse was calculated to be about 2.19 nJ. While this value is slightly above the energy consumed by biological synapses (typically ranging from 10 fJ to a few nJ) [35], it is expected that further miniaturization could significantly reduce energy consumption into the biologically relevant range. The forgetting process was triggered by terminating UV exposure, and EPSC decay was measured over time. This learning and forgetting sequence was repeated twice to assess temporal dynamics and retention characteristics across different Al doping levels. During the first learning process (Figure 5a), EPSC steadily increased with successive UV pulses, reaching a maximum at 2.0 wt% Al, indicating optimal synaptic potentiation. Furthermore, the number of pulses required to reach peak EPSC gradually increased from 40 to 42 as Al content exceeded 2.0 wt%, suggesting that moderate Al doping enhances synaptic strengthening efficiency. The peak EPSC values at each Al concentration, with the maximum (0.138 mA) at 2.0 wt%, are also plotted in Figure 5a. In the first forgetting process (Figure 5b), the time required for EPSC to decay to 70% of its peak value increased with Al content, peaking at 32 s for 2.0 wt%. This extended decay time implies improved memory retention owing to improved charge trapping and suppressed recombination. The corresponding forgetting durations for each Al concentration are plotted in Figure 5e. The second learning process (Figure 5c) was initiated from the respective forgetting thresholds (indicated by the horizontal black, red, green, blue and cyan dashed lines), and all devices required only ~25 pulses to regain their maximum EPSC levels, regardless of Al concentration. This number is significantly lower than the 40–42 pulses needed in the first learning cycle, indicating more efficient relearning due to residual carrier accumulation. This observation aligns with the pulse count trend in Figure 5a,c, highlighting the cumulative effect of repeated stimulation. As shown in Figure 5d, the second forgetting process exhibited similar or even longer retention times compared to the first cycle. Figure 5e demonstrates that the forgetting duration increases after the second learning in all Al-doped samples, indicating enhanced memory retention. Notably, the undoped ZnO device showed an increase of ∼2 s (13 s → 15 s), whereas the 2.0 wt% Al-doped AZO device showed the most significant increase (32 s → 36 s), reinforcing the idea that repetitive learning enhances long-term memory consolidation. These findings confirm that tuning the Al concentration enables precise control over both short- and long-term plasticity in AZO synaptic devices, and that repeated optical stimulation reinforces memory via cumulative charge retention and persistent photoconductivity. To further quantify the forgetting behavior observed in Figure 5a–d, Wickelgren’s power law, a widely accepted model for biological memory decay, was applied, defined as follows [11,36,37]:

I = λ × (1 + βt)^−Ψ^ (3)

where I is the memory intensity, t is the decay time, λ is the initial memory strength (i.e., the long-term memory value at t = 0 s), β is a time-scaling factor, and Ψ is the forgetting rate. Figure 5f,g display the extracted fitting parameters (λ and Ψ) as a function of Al composition. As shown in Figure 5f, λ increases with Al doping in both learning cycles, peaking at 2.0 wt%, confirming that Al incorporation facilitates charge accumulation and improves initial synaptic response by enhancing carrier separation and transport. Conversely, Figure 5g shows that the forgetting rate Ψ systematically decreases with increasing Al content up to 2.0 wt%, reaching its lowest value at this concentration. The reduced Ψ implies slower EPSC decay and enhanced memory retention. This behavior is attributed to the optimal formation of defect-mediated trap states, such as Al–oxygen vacancy complexes, that prolong photocarrier lifetime and stabilize synaptic memory [26]. However, when the Al content exceeds 2.0 wt%, Ψ increases sharply. This reversal is likely due to excessive Al incorporation, which introduces structural disorder and non-radiative recombination centers that degrade charge retention and accelerate EPSC decay. Notably, the second forgetting cycle consistently shows lower Ψ values than the first across all Al concentrations, further demonstrating the effect of repeated stimulation in reinforcing synaptic stability. These quantitative results, based on power-law fitting, are consistent with the experimental EPSC trends in Figure 5a–d and demonstrate that Al doping in AZO films is an effective strategy for improving both memory formation and retention in optoelectronic synaptic systems.

### 3.6. Visual Memory Simulation and Long-Term Retention Behavior in AZO Optoelectronic Synaptic Device Arrays

**Figure 6:**
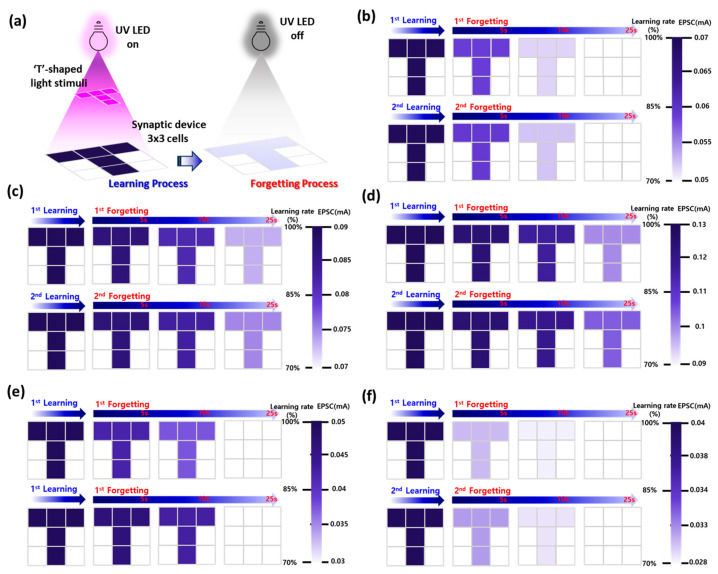
Visual memory simulation of AZO optoelectronic synaptic devices under patterned UV stimulation. (**a**) Schematic illustration of selective optical stimulation using a T-shaped UV light pattern projected onto a 3 × 3 array of AZO synaptic devices to simulate spatial memory formation and natural forgetting. Evolution of visual memory states for AZO devices with varying Al compositions: (**b**) 0 wt%, (**c**) 1.0 wt%, (**d**) 2.0 wt%, (**e**) 3.0 wt%, and (**f**) 4.0 wt%. The measured EPSC values were recorded after optical learning and mapped to grayscale intensity, with darker pixels representing stronger memory retention.

A 3 × 3 pixel array was used to simulate visual memory and assess the spatial learning behavior of AZO-based optoelectronic synaptic devices. A T-shaped UV light pattern was projected onto the device array to selectively stimulate specific pixels, thereby mimicking a spatially resolved optical learning process, as shown in Figure 6a. After the application of 100 UV light pulses (pulse width: 0.1 s, duty cycle: 50%), the devices underwent a natural forgetting phase, during which the UV illumination was removed. The resulting EPSC values of each pixel were mapped to grayscale intensities, where darker pixels indicated stronger memory states corresponding to higher EPSC retention. As shown in the comparative results across different Al doping concentrations (0~4.0 wt%), the degree of memory retention exhibited clear doping dependence. After the first learning–forgetting cycle, the pristine (0 wt%) ZnO devices showed rapid EPSC decay, with most pixels fading to near-background intensity within 25 s, as shown in Figure 6b. In contrast, the 2.0 wt% Al-doped AZO devices maintained significantly darker pixel intensities over the same duration, indicating superior retention behavior and a markedly prolonged PPC response, as shown in Figure 6d. This darker pixel persistence at 25 s becomes more prominent with increasing Al doping up to 2.0 wt% (Figure 6c,d), whereas further doping to 3.0 and 4.0 wt% (Figure 6e,f) results in complete fading of the learned pixels below the memory threshold, suggesting insufficient retention capacity at higher doping levels.

This enhanced long-term memory (LTM) performance at 2.0 wt% is attributed to the optimized formation of Al-induced trap states, particularly Al–oxygen vacancy complexes, which act as deep-level traps capable of confining photocarriers for extended periods [26]. The DX-like center model is schematically illustrated in Figure 2f as a conceptual representation based on prior reports on donor-like traps in AZO systems [26,27,28], and not as an experimentally resolved energy level in this work. At this doping level, carrier concentration and crystallinity are well-balanced: free electron density is sufficiently increased to enhance conductivity, while structural disorder remains limited, allowing effective carrier transport and long-lived trapping without excessive recombination loss. This unique combination enables more stable synaptic weights and slower EPSC decay. In the second learning and forgetting process, repetitive stimulation led to further enhancement in memory retention across all doping levels, mimicking the biological phenomenon of memory reinforcement. Notably, the second cycle showed stronger retention than the first, particularly in the 2.0 wt% device, where more pixels retained their intensity over time, indicating cumulative learning effects and strengthened synaptic memory. These findings clearly demonstrate that, by modulating Al doping concentration in AZO thin films, both the learning efficiency and forgetting dynamics of neuromorphic optoelectronic synaptic devices can be precisely controlled. In particular, 2.0 wt% Al doping provides an optimal material configuration for achieving high memory fidelity, extended PPC, and robust visual memory performance, underscoring the critical role of defect engineering in artificial synapse design.

## 4. Conclusions

This study presents a systematic investigation of Al-doped ZnO thin films synthesized using a sol–gel method for use in optoelectronic synaptic devices. By varying Al concentration from 0 to 4.0 wt%, we explored its effects on structural, optical, and synaptic properties. The results show that moderate Al doping effectively enhances carrier concentration, photoconductivity, and synaptic plasticity by optimizing crystallinity and defect states. Although direct chemical state analysis via XPS or EDX was not performed in this study, the successful incorporation of Al was indirectly confirmed through Hall effect measurements, which showed a substantial increase in carrier concentration from 3.1 × 10^17^ cm^−3^ (undoped) to 2.2 × 10^18^ cm^−3^ at 2.0 wt% Al. This enhancement supports the substitutional incorporation of Al^3+^ ions into the Zn^2+^ sites, acting as electron donors. The formation of Al–oxygen vacancy complexes was found to play a role in enabling PPC and memory retention. Consequently, the 2.0 wt% AZO devices demonstrated superior LTM performance, particularly exhibiting longer retention times and slower EPSC decay, as confirmed by EPSC-based forgetting analysis and 3 × 3 visual memory simulations. Visual encoding using patterned UV input showed that 2.0 wt% devices maintained the strongest pixel-level memory retention during repeated learning–forgetting cycles. These results highlight the importance of defect and doping control in oxide semiconductors for neuromorphic applications. The observed PPC and memory retention behavior closely mimic biological synapses and support low-power, light-driven learning processes. Thus, sol–gel-derived AZO devices present a potential scalable and cost-effective approach for future in-sensor computing and neuromorphic vision systems, although further studies on large-area uniformity and process integration are needed to fully establish their manufacturability. Material-level engineering in such systems enables high-efficiency optical learning and memory functions. Nevertheless, the current study is limited to UV stimulation and a relatively simple device geometry. Future work should explore broadband or visible-light operation, miniaturization of pixel arrays, and integration with complementary logic circuits to further validate system-level neuromorphic functionality.

## Data Availability

The data presented in this study are available on request from the corresponding author. The data are not publicly available due to privacy concerns.
